# Enhancing the experience and outcomes of children with complex care needs in acute paediatric settings: a realist review protocol

**DOI:** 10.1136/bmjopen-2024-097328

**Published:** 2025-03-12

**Authors:** Emma Popejoy, Jane Coad, Eyal Cohen, Alison Pearson, Rachel Williams, Joseph C Manning

**Affiliations:** 1 Nottingham Children’s Hospital, Nottingham University Hospitals NHS Trust, Nottingham, UK; 2 School of Healthcare, University of Leicester, Leicester, UK; 3 Centre for Children and Young Peoples Health Research, University of Nottingham School of Health Sciences, Nottingham, UK; 4 Centre for Care Excellence, University Hospitals Coventry and Warwickshire, Coventry, UK; 5 Pediatrics, Hospital for Sick Kids, Toronto, Ontario, Canada; 6 SickKids Research Institute, Toronto, Ontario, Canada; 7 Institute of Medical Science, University of Toronto, Toronto, Ontario, Canada; 8 University of Exeter, Exeter, UK; 9 Nottingham University Hospitals NHS Trust, Nottingham, UK

**Keywords:** PAEDIATRICS, Hospitals, Review

## Abstract

**Abstract:**

**Introduction:**

The number of babies, children and young people with complex care needs (henceforth children with complex care needs (CCCN)) in England has increased in recent decades, and this has also been recognised globally. CCCN may have frequent and lengthy hospital admissions, but during these episodes, their needs are not always met, potentially resulting in suboptimal experiences and outcomes. Despite increased numbers of CCCN accessing acute care and displaying greater complexity, much of the contemporary literature has focused on primary care coordination between health, education and social care. Research specifically focused on CCCN in the acute care setting is largely absent. This realist review aims to understand how optimal experience and outcomes are achieved for CCCN during acute care, in different settings, for whom and why.

**Methods and analysis:**

This realist review will proceed through six steps: (1) clarifying the scope of the review, (2) searching for evidence, (3) data selection and quality appraisal, (4) data extraction, (5) analysis and synthesis and (6) dissemination. We will search Medline, Cumulated Index in Nursing and Allied Health Literature and PsycINFO, alongside grey literature and other sources and will carry out citation tracking. Patient and public involvement and engagement have aided in the development of this protocol and will be maintained through regular consultations with a stakeholder group throughout the review. The review will result in a programme theory which will include context-mechanism-outcome configurations and provide data to support claims of generative causation.

**Ethics and dissemination:**

Ethical approval is not required for this review as it does not involve primary research. The programme theory developed will be disseminated through peer-reviewed publications and relevant conferences. It will subsequently inform the development of an intervention to improve acute care for CCCN.

**PROSPERO registration number:**

CRD42024591231.

**STRENGTHS AND LIMITATIONS OF THIS STUDY**

- An initial programme theory has already been developed through reference to formal theory and feedback provided during patient and public involvement and engagement activity.
- A stakeholder group will be consulted throughout the review to ensure that the review focuses on the key aspects important to them and is informed by their expertise and experience.
- The scope of the review will be determined as the review progresses, and some areas of literature may need to be prioritised over others, meaning that not all areas will be interrogated.

## Introduction

 The number of babies, children and young people (BCYP) with complex care needs, henceforth referred to as children with complex care needs (CCCN), has increased markedly over recent decades.^1^ This phenomenon is a result of improvements in survival of premature infants and those with chronic conditions and disabilities.^2^

Complex care needs in children ‘refer to multidimensional health and social care needs in the presence of a recognized medical condition or where there is no unifying diagnosis. They are individual and contextualized, are continuing and dynamic, and are present across a range of settings, impacted by healthcare structure’^1^[p1647]. A variety of terms are used both clinically and in the literature to describe CCCN. An overlapping and widely accepted term is children with medical complexity who have been identified as having four attributes: a complex chronic condition, functional limitations, high healthcare use and substantial care needs.^2^

Some CCCN will have multiple long-term conditions, whereas others will have a single condition which impacts various body systems, resulting in interaction with multiple parts of the healthcare system and thus complexity. The National Institute for Health and Care Research (NIHR; the funder) has designated research into multiple long-term conditions and single-condition complex care needs as a key priority.^3^ The NIHR uses the term complex care needs^4^ and therefore it is the term which will be used within this protocol. To operationalise the term for the purpose of undertaking this review, the four attributes of medical complexity (described above) will be used.^2^

### Prevalence of CCCN

Accurate international prevalence data for children with disabilities and complex care needs are lacking, largely due to heterogeneity in definitions and methods of estimation.^5^ A Canadian study which examined the prevalence of CCCN in Ontario, as ascertained through hospitalisations, found that approximately 0.67% of the paediatric population have complex care needs.^6^ The authors state that this is likely to be an underestimate, as CCCN not hospitalised during the study period would not have been included.^6^ A study conducted in the USA estimated that in 2016–2017, 1.6% of US children had complex care needs.^7^

In England, data on the prevalence of CCCN are inadequate, as highlighted by the Royal College of Paediatrics and Child Health; ‘we do not even know how many children in this country have a disability, who these children are, and which services they are known to’^8^[p18]. Studies investigating prevalence in adjacent or overlapping populations can provide a guide to the prevalence of CCCN in England. A small multimorbidity study found that approximately 4.7% of children had two or more long-term conditions,^9^ and a national prevalence study investigating life-limiting conditions (LLC) and life-threatening conditions (LTC) identified the prevalence of children living with LLC and LTC in England in 2018 to be 0.66%, with this number projected to rise further.^10^ Many BCYP from these respective studies are likely to have complex care needs, yet the exact population of this particular group of BCYP is unknown.

### Acute care for CCCN

CCCN have frequent and lengthy hospital admissions, including admission to the paediatric intensive care unit.^11 12^ During hospital admissions, CCCN are at higher risk than other children in terms of experiencing errors, such as medication incidents and device-related complications.^13^,^16^ The acute care environment is also not a favourable setting to promote the physical and social development of young CCCN.^17^ Evidence also exists which suggests that many families of CCCN have poor experiences of, and adverse social outcomes related to, care.^18^,^20^

While the population of CCCN represents small absolute numbers,^10^ they are associated with extremely high healthcare costs.^21^ Punjabi *et al*^21^ identified that children in the top 5% of healthcare expenditure accounted for more than 50% overall paediatric healthcare costs, and that 62.7% of their healthcare spending was attributable to inpatient costs. Children with multiple morbidities had significantly higher costs than those with none or with one chronic condition.^21^ Both the social and economic case for improving acute care for this cohort of patients is, therefore, evident.

### Current knowledge and gaps

In recent years, there has been a focus on multiple long-term conditions and CCCN, with an increasing number of documents related to this population published over the previous few decades.^22^ Within the UK, the NIHR has identified multiple long-term conditions and CCCN as a key priority for research.^23^ There has also been growth in international research focused on CCCN, with specific attention to coordination of care across a variety of organisational and setting boundaries. A recent Cochrane systematic review^24^ aimed to assess the effectiveness of a variety of comprehensive care programmes for CCCN across different health and care settings, but only four studies were eligible for inclusion. Variability in eligibility criteria and reporting of outcomes meant that there was insufficient evidence to draw firm conclusions about the effectiveness of these programmes.^24^ A scoping review of outcomes research for CCCN identified that the most frequently reported outcomes are related to economic considerations, for example, emergency department (ED) visits and hospital admissions, rather than focusing on ‘humanistic’ outcomes which are meaningful to families.^25^ Such economic outcomes do not demonstrate the final outcomes of acute care, and the multiple impacts of acute care on the child and family must be considered.

A recent scoping review of the inpatient experiences of families of CCCN identified partnership as a key theme and the ideal descriptor of inpatient care, yet found that families experienced barriers to partnership which resulted in poor outcomes or experiences.^26^ The authors of this review also highlighted a lack of acute care-focused interventional research for CCCN,^26^ which was similarly identified by White *et al,* 7 years prior.^27^ Furthermore, last year, a James Lind Alliance Priority Setting Partnership recognised the need for improvement in inpatient care for CCCN as its number one priority.^28^ CCCN are frequent users of acute care services,^29^ and, as outlined above, these services are associated with significant social disadvantage for CCCN and their families, and financial costs to the health service. Despite growing research focused on care coordination for CCCN, to date, their acute care experiences and outcomes have been neglected. It is therefore imperative that a thorough investigation is conducted imminently to determine the existing acute care interventions for CCCN and the impact that these have on families’ experiences and outcomes.

CCCN are a heterogeneous population accessing a vast array of acute care settings with different contexts, specialities and experiences of caring for CCCN.^30^ Acute care interventions for CCCN are therefore likely to vary in the experiences and outcomes generated, based on the different settings in which they are implemented. Using a realist approach to review the current evidence will facilitate the development of explanations of how and why the interventions work, or do not work, in relation to the specific contexts of their implementation.^31 32^ Such an approach has been advocated by Berry and Feudtner^33^ who argue that, to improve care for CCCN, it is essential to understand the mechanisms through which interventions result in improved outcomes, so that programmes can be effectively designed and modified, based on the health service structure and other relevant variables.

This review aims to address these contemporary evidence gaps by using a realist approach to build understanding of how and why interventions work, or do not work, in relation to the context in which they are implemented. Following on from the Cochrane systematic review^24^ which was largely inconclusive regarding the effectiveness of care coordination strategies and the scoping review^26^ which identified the ideal description of inpatient care and how experiences deviated from this, this realist review will extend the current knowledge by providing an explanation of how and why acute care interventions for CCCN result in their associated outcomes and experience.

The specific review questions are:

1. What are the experiences and important outcomes of acute care interventions for CCCN and their families?

2. What are the mechanisms through which experiences and outcomes are generated by acute care interventions for CCCN and their families?

3. What influence does the setting and context have on the activation of mechanisms in acute care interventions for CCCN and their families?

## Methods and analysis

Realist reviews are theory-driven approaches to evidence synthesis which aim to provide coherent explanations of the influence of different contexts on outcomes. Realist reviews focus on identifying causation and result in a programme theory made up of context + mechanism = outcome configurations (CMOCs).^31^ Contexts are pre-existing structures which may influence a mechanism, such as individuals (their characteristics and capacities), interpersonal relations, institutional settings (their rules and norms), and infrastructure (the economic, cultural and social setting).^34^ Mechanisms refer to the causal process through which outcomes of interest are generated and these are context dependent.^35^

Realist reviews provide a method of reviewing and synthesising the evidence of complex interventions^36^ and go beyond identifying whether an intervention works, thus providing utility for implementation of the intervention. Realist reviews offer explanations about how and why the intervention works (or does not) and the contextual factors which mediate the outcomes.^31^ Realist reviews recognise that outcomes result from the interaction between interventions and contexts^31^ and, using generative causation, help to explain how and why they work, or not, in particular settings.^32^

Interventions to improve acute care for CCCN are likely to be considered ‘complex’, as they involve multiple components, target various behaviours and require expertise and skills to deliver them.^37^ CCCN are a heterogeneous population, cared for across vast regions and services^38^ and who often experience inequity in service provision.^39^ This contextual complexity demands a review approach which is sensitive to recognise and explain the differences in outcomes based on the setting and, therefore, the activation of mechanisms. A realist review will be undertaken to understand how optimal outcomes and experience are achieved for CCCN and their families during acute care, in different settings, for whom and why.

### Stakeholder group

A stakeholder group will be established, and it will be a diverse group of approximately 10–15 children and young people (where possible), parent/carers, healthcare professionals, National Health Service leaders and policymakers with experience of acute care for CCCN. Potential stakeholder group members will be identified through the project team and their wider networks. They will provide direction to the study team through four workshops/meetings throughout the review and act in an advisory capacity to the core team undertaking the review. They will provide advice and direction regarding the focus of the review, signposting to potential sources of evidence, sense-checking the findings and guidance for dissemination. The stakeholder group will be facilitated by researchers with expertise in working with CCCN and patient and public involvement and engagement (PPIE) who will, at the first meeting, provide clear ground rules, outline the methods of the review and the role of the stakeholder group. They will ensure that during every stakeholder group meeting, the opportunity is provided for each member to share their views and experiences.

### Review

This review will conform to the Realist And Meta-narrative Evidence Syntheses – Evolving Standards publication standards for realist reviews^40^ and will proceed through the six steps as described by Pawson.^41^

#### Step 1: clarifying the scope and review question

**Figure 1:**
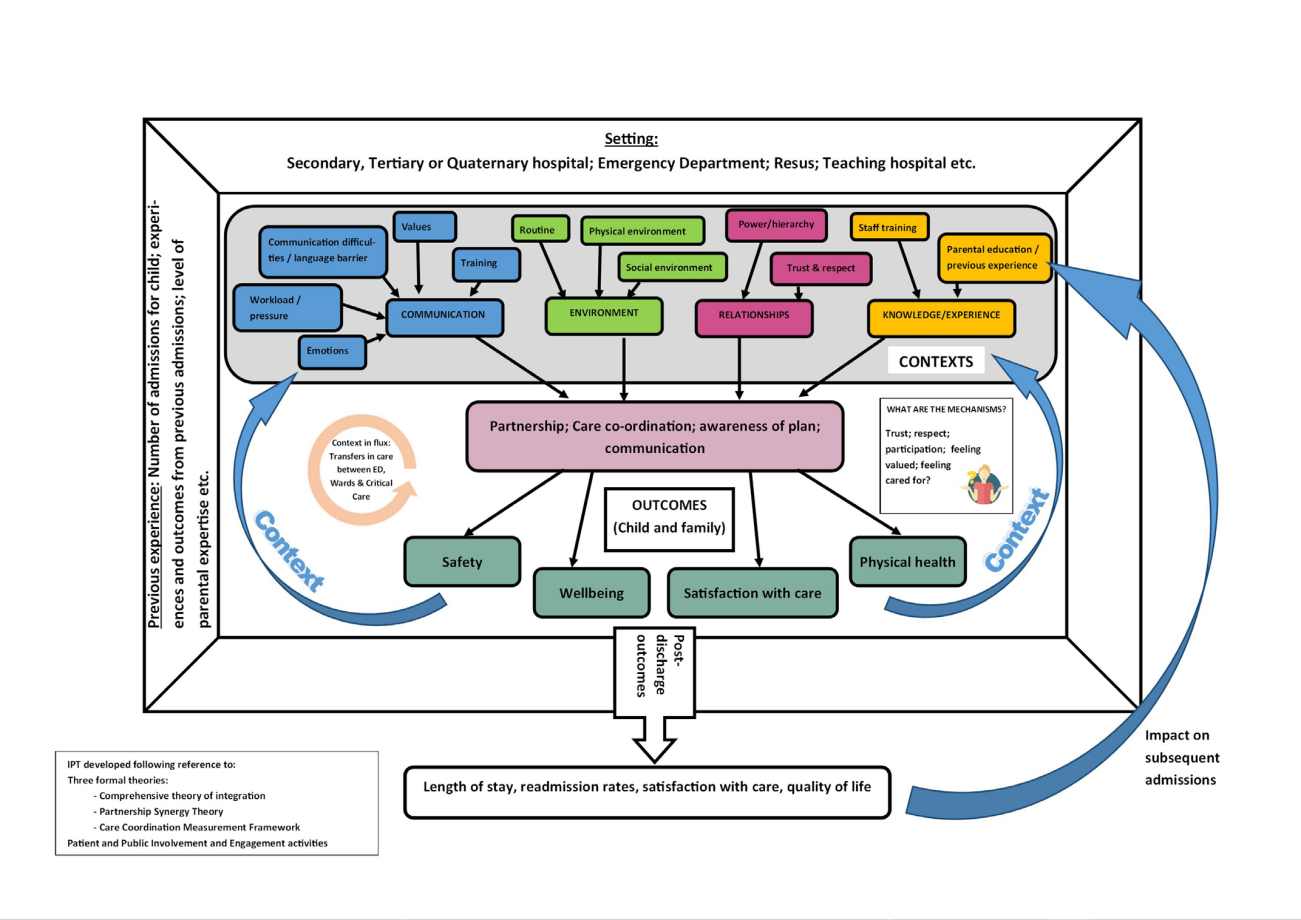
Initial programme theory.

Preliminary searching identified three theories and frameworks (comprehensive theory of integration,^42^ partnership synergy theory^43^ and care coordination measurement framework^44^) which will likely be helpful in understanding how and why acute care interventions for CCCN work to deliver optimal outcomes, or not. These have therefore informed the initial programme theory (figure 1), as has PPIE work undertaken in the development of this protocol. The initial programme theory, which was drafted by EP and refined through discussions with the other authors and PPIE advisors, will help to guide the searches and synthesis.

The scope of the review and specific questions to be answered will be clarified over the first few months of the review and proceed iteratively as the initial searches are conducted. Informal literature searching will be conducted to identify and map any existing programme theories which may help to develop and refine the initial programme theory.

The review focus and questions will be refined through an iterative process of consultation with the stakeholder group. Stakeholder group workshops will guide the focus of the review through in-depth discussions of the existing evidence with the study team and based on their own experiences. Any amendments to this protocol will be documented on PROSPERO.

#### Step 2: searching for evidence

Pawson^41^ suggests this step begins with a background search to fully comprehend the breadth of the existing evidence and to identify whether there is likely to be sufficient relevant material to answer the review question. In planning for this review, some initial preliminary searches have been conducted, and these indicate that the questions that the review proposes to ask should be answerable within the existing body of evidence. An example search strategy is provided (online supplemental material).

It is anticipated that the majority of the evidence within this review will be from published primary or secondary research; however, it is likely that other sources including grey literature, policy documents and media, such as blogs or podcasts, may also be included. Within realist reviews, evidence searching aims to identify any sources which may inform the developing programme theory.^45^ The inclusion of grey literature, policy documents and media may provide data to support the developing programme theory and enhance our understanding of the relevant contexts, mechanisms and outcomes, which may not be uncovered through reference to published research alone. For brevity, the term documents will be used here to refer to any type of evidence to be included in the review.

The search for empirical evidence aims to identify evidence which informs or tests the initial programme theory or rather some ‘precise set of assumptions held in the programme theory’^41^[p78]. Specific attention during the initial searches will be paid to any underlying, pre-existing programme theories, including those located in policy or legislative documents.^36^ For this review, such documents may include policies relating to multimorbidity and physical or intellectual disabilities. Searches will be conducted iteratively, informed by discussions with the information specialist (RW). Searches will be modified and complementary searches undertaken as our understanding of the programme theory evolves and as additional evidence to support emerging or refined aspects of the theory is required.^46^ A range of databases (Cumulated Index in Nursing and Allied Health Literature, PsycINFO and Medline) will be searched and have been chosen for their focus on clinical care in medicine, nursing and allied healthcare and behavioural sciences. Grey literature will be searched using OpenGrey, Health Management Information Consortium, NICE and Google Scholar. No restrictions will be imposed on the searches based on the date of publication. Databases will be searched using keywords related to the three key areas: CCCN, emergency care and inpatient care. Media sources will be identified through internet search engines. The research team and stakeholder group members will also identify any relevant documents for inclusion. Initial search terms will be expanded to include different bodies of literature as required, thus providing evidence on specific aspects of the theory being tested. However, preliminary searches indicate that such additional and broader searches may not be required. Included documents will undergo citation screening. Inclusion and exclusion criteria will be broad as outlined in table 1.

**Table 1:** Review inclusion and exclusion criteria

Inclusion criteria	
Population	CCCN ≤18 years.Parent/carers of CCCN ≤18 years.Healthcare Professionals caring for CCCN ≤18 years.
Interventionorphenomenon of interest	Any programme which aims to improve the experiences of, and outcomes related to, acute care (delivered by any acute care provider) for CCCN and their families.Experiences of, and outcomes related to, acute care (delivered by any acute care provider) for CCCN and their families.
Outcomes	Outcomes of interest will not be specified in advance, but may include outcomes related to physical, social and emotional well-being, experience of care, length of stay, readmissions and numbers of complaints. These outcomes may be described through validated tools or qualitative methods.
Source type	No restriction on study design.Grey literature, policy documents, opinion pieces, media, for example, podcasts, and any other sources which can shed light on the phenomenon of interest will be included.
**Exclusion criteria**Documents not published in English.Documents related to those with complex care needs >18 years, unless data for CCCN are presented separately.Documents related to care for CCCN in the community, within the third sector or focused on care coordination across the health, social and education care continuum, unless these contain aspects specific to improving acute care.	

The iterative nature of realist reviews demands that inclusion and exclusion criteria are revised during the review in light of the evolving focus and findings. This table outlines criteria which have been determined prior to immersion in the evidence. Documents fulfilling exclusion criteria may be included in the later stages of the review to provide evidence when developing or testing the programme theory.

CCCNchildren with complex care needs

Search results will be screened independently by one reviewer, with a random sample of 10% dual screened, to ensure that inclusion criteria are applied consistently, at both the ‘Title and Abstract’ and ‘Full Text’ screening stages. A Kappa score will be calculated to demonstrate the level of agreement and reliability in applying the inclusion and exclusion criteria. Disagreements will be resolved between the two reviewers where possible or resolved through discussion with the wider study team where necessary.

Subsequent searches to refine and test the programme theory will be decided on purposively following the initial searches. It may be necessary and appropriate to broaden the search to include research focused on an overlapping group of children, for example, those with intellectual disability, when testing the programme theory for coherence and plausibility. Any such decisions regarding search strategy, inclusion criteria and when to stop searching will be considered regularly with the information specialist and the wider study team, and an audit trail will be kept. Further searching will be terminated when the programme theory is considered plausible and coherent^46^ by the study team and stakeholder group, and no further evidence adds to theory development.

#### Step 3: data selection and quality appraisal

The realist approach to quality appraisal considers the quality of the included document based on the part of it which informs the developing programme theory.^41^ Instead of undertaking formalised quality appraisals, realist reviews focus on the concepts of relevance and rigour in the inclusion of documents for review.^40^ Relevance considers whether the document can aid in the development or testing of the programme theory, and rigour refers to the trustworthiness and credibility of the research. Rigour may be informed by the application of existing methodological appraisal checklists, where appropriate, but will primarily focus on assessing and explicitly recording how each document contributes to the development of the programme theory.^45 47 48^

Document selection will be iterative, and a document excluded at the start of the review may be included at a later stage if it is relevant to some aspect of theory development or testing. Rayyan, an online review software, will be used to aid document selection and cataloguing. Decisions about document selection will be recorded using a Consolidated Standards of Reporting Trials (CONSORT) diagram in order to provide traceability. This will be included in the study outputs.

#### Step 4: data extraction

Although represented as distinct phases, data extraction will occur concurrently with document selection. The team will employ NVivo and Microsoft Excel in the extraction and synthesis to highlight and annotate the documents and provide an audit trail of their usage. Documents will be coded within NVivo by one reviewer and, similar to data selection, a random subsample of 10% sources will be independently checked for consistency. Any major differences in coding will be discussed and resolved by the two reviewers, or through reference to the wider study team, where required. Coding will aim to identify major ‘themes’ in relation to the experiences and outcomes of acute care for CCCN. It will be both deductive, through reference to the initial programme theory (figure 1), and inductive to account for themes not previously considered.

Data extraction in realist reviews is specific to the individual document, and no single data extraction form is likely to be appropriate. Rather, a suite of bespoke forms will be developed to assist in the extraction of data.^49^ These will catalogue the document aims, methods, participants, details of any intervention, outcomes measured and will provide an indication of which aspect of the programme theory the data relates to.

#### Step 5: analysis and synthesis

The analytical task in realist reviews is to identify evidence of generative causation which links the context of an intervention to the mechanisms enacted and subsequently outcomes achieved.^46^ The aim is to move from the initial programme theory to a refined programme theory which explains how and why an intervention works, or does not work, in a given setting, through reference to the included documents.

The extracted data will be themed, and the contents of each theme interrogated to identify relevant contexts, mechanisms and outcomes and how these relate to each other. Possible CMOCs will be developed within each theme to provide an explanatory account of how the outcomes for CCCN are achieved in the acute care setting, paying particular attention to any differences in CMOCs depending on the type of setting and its features. Where CMOCs are incomplete, additional searches will be undertaken to search for the contexts, mechanisms or outcomes which may be missing or to clarify and refine the explanatory programme theory. CMOCs will be assessed for plausibility and coherence by the review team and stakeholder group. Rival CMOCs will be adjudicated between by assessing the evidence on which they were developed, with specific attention to their relevance and rigour. Where this assessment fails to clearly identify a stronger CMOC, further evidence will be sought in support of the CMOCs until sufficient evidence is gathered to provide the review team with confidence that one CMOC is more convincing. The resulting CMOCs will be situated within the wider programme theory.

#### Step 6: dissemination

This review and the resultant programme theory will be disseminated through peer-reviewed publication and presentation at relevant conferences. It will also be shared through national networks for clinicians working with CCCN.

As expressed by Pawson, ‘realist reviews deliver models, which in policy terms are not the end but the beginning of the story’^41^(p100) and can be used to inform the development of interventions.^50^ The programme theory resulting from this realist review will be used to inform the development of an intervention to improve acute care for CCCN. A subsequent phase of this project will involve a series of focus groups with key stakeholders to develop the intervention using an experience-based co-design approach,^51^ which will then proceed to feasibility testing and realist evaluation.

### Patient and public involvement and engagement

PPIE has been sought in the development of this review protocol and a wider study proposal which aims to develop an acute care intervention for CCCN. To date, 20 diverse families of CCCN have been consulted, and all have highlighted improving acute care for CCCN as a key research priority. The families consulted represent children of various ages and a range of diagnoses and are geographically and ethnically diverse. The focus of the project has evolved through PPIE, from a sole focus on inpatient care to acute care, with the inclusion of ED attendance. Following discussion with numerous families, it was highlighted that the ED is a key area of concern for them during acute admissions.

The stakeholder group will include children and young people (where possible) and parent/carers, alongside healthcare professionals and policy makers. The study team will meet regularly with the stakeholder group during the review and therefore ensure that PPIE is maintained throughout. Virtual meetings will be held and will cover the content of the programme theory, the coherence and plausibility of the evolving programme theory and possible sources of evidence.^45^ We aim to ensure diversity in the stakeholder group so that it represents the spectrum of families of CCCN accessing acute care. To date, effort has been made to gain representation from the diverse cultural, social and ethnic backgrounds of CCCN, and particular attention has been paid to ensuring representation from groups identified as having higher prevalence.^10 52^ This review protocol has been coauthored by a parent of a child with CCCN (AP) who has experience in realist methods, and she will be part of the study advisory group.

## Ethics and dissemination

Ethical approval will not be required, as this is a literature review and does not involve primary data collection. As previously identified, this review will be published in a peer-reviewed journal and presented at relevant conferences. It will subsequently inform the development of an intervention to improve acute care for CCCN.

## supplementary material

10.1136/bmjopen-2024-097328 online supplemental file 1
