# Supplementary material for: Enhancing the experience and outcomes of children with complex care needs in acute paediatric settings: a realist review protocol
Source: BMJ Open. 2025 Mar 12;15(3):e097328. doi: 10.1136/bmjopen-2024-097328 (PMC11904347; doi:10.1136/bmjopen-2024-097328)
Supplement: online supplemental file 1 [file bmjopen-15-3-s001.pdf]

Ovid MEDLINE(R) ALL <1946 to July 15, 2024>

1 child* with medical complexit*.ti. or child* with medical complexit*.ab. 610

2 (child* with special health care need* or CSHCN).ti. or (child* with special health care need* or CSHCN).ab. 1154

3 child* with complex need*.ti. or child* with complex need*.ab. 110

4 technology dependent child*.ti. or technology dependent child*.ab. 137

5 medically fragile child*.ti. or medically fragile child*.ab. 69

6 child* with special needs.ti. or child* with special needs.ab. 761

7 1 or 2 or 3 or 4 or 5 or 6 2750

8 ((child or children or infant* or teenager* or adolescent* or p?ediatric) adj4 (medical complexity or medically fragile or complex need* or special need* or special health care need* or multi-morbidit* or complex chronic condition* or complex medical condition* or life-limiting condition* or rare disease*)).ti. or ((child or children or infant* or teenager* or adolescent* or p?ediatric) adj4 (medical complexity or medically fragile or complex need* or special need* or special health care need* or multi-morbidit* or complex chronic condition* or complex medical condition* or life-limiting condition* or rare disease*)).ab. 5293

9 (life-limiting condition* adj4 (child or children or infant* or teenager* or adolescent* or p?ediatric)).ti. or (life-limiting condition* adj4 (child or children or infant* or teenager* or adolescent* or p?ediatric)).ab. 300

10 *adolescent, hospitalized/ or *child, hospitalized/ or *inpatients/ 19161

11 *secondary care/ or *tertiary healthcare/ 879

12 (secondary care or tertiary care or intensive care or critical care or emergency care or emergency department or urgent care or inpatient* or hospitali#ation).ti. or (secondary care or tertiary care or intensive care or critical care or emergency care or emergency department or urgent care or inpatient* or hospitali#ation).ab. 715629

13 (hospital* adj4 (stay or admission or admitted)).ti. or (hospital* adj4 (stay or admission or admitted)).ab. 245864

14 10 or 11 or 12 or 13 893905

15 7 or 8 or 9 5479

16 14 and 15 795

17 (neonat* or newborn or NICU or preterm).ti. 237060

18 16 not 17 756
