# Supplementary material for: Enhancing the experience and outcomes of children with complex care needs in acute paediatric settings: a realist review protocol
Source: BMJ Open. 2025 Mar 12;15(3):e097328. doi: 10.1136/bmjopen-2024-097328 (PMC11904347; doi:10.1136/bmjopen-2024-097328)
Supplement: Supplementary file 2 [file bmjopen-2024-097328.reviewer_comments.pdf]

PEER REVIEW HISTORY

BMJ Open publishes all reviews undertaken for accepted manuscripts. Reviewers are asked to complete a checklist review form and are provided with free text boxes to elaborate on their assessment. These free text comments are reproduced below.

ARTICLE DETAILS

Title (Provisional)

Enhancing the experience and outcomes of children with complex care needs in acute paediatric settings: a realist review protocol

Authors

Popejoy, Emma; Coad, Jane; Cohen, Eyal; Pearson, Alison; Williams, Rachel; Manning, Joseph C

VERSION 1 - REVIEW

**Reviewer**	**1**
**Name**	**Atout, Maha**
**Affiliation**	**Philadelphia University, nursing**
**Date**	**26-Dec-2024**
**COI**	**None**

Thank you for inviting me to review this protocol entitled Enhancing the experience and outcomes of children with complex care needs in acute paediatric settings: what works, for whom, and under what circumstances, a realist review protocol.”.

This protocol, in my opinion, is well-written and should provide valuable insights into achieving optimal experiences and outcomes for CCCN in various acute care settings. It is an underresearched area in the literature, and the current review will provide valuable findings for a future intervention to improve the experiences and outcomes of children with complex care needs in acute paediatric settings.

Some minor suggestions:

• Page 4, Line 28-32: reference needed

• Lines 58-60 on page 5 require a reference.

• Page 8: lines 3-6. Please justify using sources such as grey literature, etc.

• Some old references may need renewing.

**Reviewer**	**2**
**Name**	**Jerofke-Owen, Teresa**
**Affiliation**	**Marquette University - College of Nursing**
**Date**	**13-Jan-2025**
**COI**	**None**

This realist review has the potential to fill a huge gap in the literature. I have some suggestions for clarity and also some suggestions when looking at your initial programme theory. Please see attached document. Thank you

Thank you for the opportunity to review this protocol. This realist review aims to fill a large gap in research for CCCN. I have some suggestions for clarity.

Please be more specific about how you anticipate that the stakeholder group will provide direction. How many do you anticipate to be in this stakeholder group? What do you mean by regular workshops? How frequent? How will you manage power differentials? Obviously, there will be power differentials if you have children in the groups vs healthcare professionals, service leaders and policy makers. How will you ensure everyone will participate and be engaged? Realist reviews are complex – how will you ensure that patients/families will understand their role?

Page 7 line 29: The wording about whether interventions deliver outcomes….I would say any intervention has some type of outcome. I think this needs to be re-worded.

Page 8 line 16: unclear what you mean that the search will be modified as your understanding of how the programme theory works grows? Should have a clear search strategy for this protocol. I can't tell from the author list – but would highly recommend a librarian on your team

Pg 8 line 32: The number of articles you may end up reviewing could be very substantial as you are not limiting to a particular time period. It may be unrealistic to have 1 sole researcher to review and abstract all the data. I've conducted and published a realist review – if you have over 100 articles to abstract data from, that is very time consuming.

Page 9 line 15: For outcomes – this doesn't have to be a specific outcome measurement tool. There are likely going to be physical, psychosocial, psychological outcomes. What about qualitative literature? There likely won't be measures used in those studies.

In your initial programme theory – seeing we are dealing with pediatric patients – there are going to be parent/caregiver outcomes and also child outcomes. Are you going to be looking at outcomes of the parent as well (in your initial programme theory you have patient satisfaction with care listed but I don't see family satisfaction specified). Also

how is the satisfaction with care in the green box different from the post-discharge outcome of patient satisfaction with care?

Within your contexts – it isn't clear which concepts are patient, family, or provider based. As you mentioned in the text – contextual factors include individual factors (would be both the family and patient), provider factors and organizational.

Are you going to be focusing on a certain type of provider or any type of intervention provided in the acute care setting by any type of provider?

In your initial programme theory under 'what are the mechanisms?' box you have 'feeling valued' and 'feeling cared for'. Aren't those outcomes? Also are you conceptualizing partnership as a mechanism or is that an outcome? There are many mechanisms by which providers engage families in the care of the pediatric patient that are not reflected in your initial programme theory. The only things labeled as mechanisms right now are 'trust', 'respect', 'participation', 'feeling valued', and 'feeling cared for'. Maybe I am misinterpreting the model?

Please be explicit about who was responsible for constructing the initial programme theory.

**Reviewer**	**3**
**Name**	**Basu, Anna**
**Affiliation**	**Newcastle University, Institute of Neuroscience**
**Date**	**14-Jan-2025**
**COI**	**None**

The authors have produced a clear protocol with well reasoned justification for the work. The nature of the methodology appears appropriate for the approach. The paper is well written and well referenced and has been informed by stakeholder input. The initial programme theory diagram is a little hard to read and my only suggestion is to optimise this - though on a full A4 page in print it may be less problematic.

VERSION 1 - AUTHOR RESPONSE

**Reviewer comments**	**Authors' Response**
**Reviewer 1**	
Page 4, Line 28-32: reference needed	Reference added.
Lines 58-60 on page 5 require a reference.	Reference added.
Page 8: lines 3-6. Please justify using sources such as grey literature, etc.	Thank you for highlighting the need to justify this further. We have restructured the paragraph and highlighted that realist searching focuses on identifying sources

	which can contribute to the developing programme theory, without reference to the traditional hierarchy of evidence.
Some old references may need renewing.	We have reviewed the reference list. Many of the older references are either theoretical or seminal work in the field of CCCN. Please advise if there are specific references that you believe require updating or are aware of references which would be valuable to include.
**Reviewer 2**	
Please be more specific about how you anticipate that the stakeholder group will provide direction. How many do you anticipate to be in this stakeholder group? What do you mean by regular workshops? How frequent? How will you manage power differentials? Obviously, there will be power differentials if you have children in the groups vs healthcare professionals, service leaders and policy makers. How will you ensure everyone will participate and be engaged? Realist reviews are complex – how will you ensure that patients/families will understand their role?	Thank you for highlighting the need to provide further information about the stakeholder group. We have provided some further detail about the numbers (10-15) of people who will make up the stakeholder group and highlighted that the meetings will be led by researchers experienced in working with CCCN and conducting PPIE. This experience will help to ensure participation and minimise the effect of power imbalance present within the group.<br>We have also now included an explanation that at the first stakeholder group meeting, ground rules, an explanation of the review process and role of the group will be provided.
Page 7 line 29: The wording about whether interventions deliver outcomes….I would say any intervention has some type of outcome. I think this needs to be re-worded.	Thank you for highlighting this. We have amended to state "optimal outcomes".
Page 8 line 16: unclear what you mean that the search will be modified as your understanding of how the programme theory works grows? Should have a clear search strategy for this protocol. I can't tell from the author list – but would highly recommend a librarian on your team	We have provided a reference (Realist Synthesis: RAMESES Training Materials) to support this statement that searching will be iterative and modified as the theoretical understanding grows. The Training Materials state: "Searching is likely to need to be iterative because, as the synthesis progresses, new or refined elements of theory may be required to explain particular findings, or to examine specific aspects of particular processes. As new elements of theory are included, searches for evidence to support, refute, or refine those elements may be required." Please advise if you feel it would be valuable to include this quote in the manuscript.<br>We will conduct the initial, and any subsequent searches in close consultation with Rachel Williams, our co-author and the librarian working on this review.

Pg 8 line 32: The number of articles you may end up reviewing could be very substantial as you are not limiting to a particular time period. It may be unrealistic to have 1 sole researcher to review and abstract all the data. I've conducted and published a realist review – if you have over 100 articles to abstract data from, that is very time consuming.	Thank you for highlighting this potential issue. From our initial scoping searches, we believe that the numbers of sources for review will not be vast and should be manageable by one reviewer working solely on the review.
Page 9 line 15: For outcomes – this doesn't have to be a specific outcome measurement tool. There are likely going to be physical, psychosocial, psychological outcomes. What about qualitative literature? There likely won't be measures used in those studies.	Thank you for raising this issue. We agree with your comment and have refined the content of the table to highlight the variety of outcomes and possible means of assessing them.
In your initial programme theory – seeing we are dealing with pediatric patients – there are going to be parent/caregiver outcomes and also child outcomes. Are you going to be looking at outcomes of the parent as well (in your initial programme theory you have patient satisfaction with care listed but I don't see family satisfaction specified). Also how is the satisfaction with care in the green box different from the post-discharge outcome of patient satisfaction with care?	Thank you for raising this point and highlighting our need to clarify the diagram further. We are absolutely interested in the outcomes of the child and their family, as reflected in the review questions. We have revised the diagram so that where it previously stated, "patient satisfaction with care", it now says "satisfaction with care", as this can refer to satisfaction from the child and/or parent's point of view. In addition, we have made it explicit that we are interested in the child and family outcomes by changing the 'Outcomes' box to 'Outcomes (Child and family)'.<br><br>The green boxes reflect satisfaction during the admission, which may be fluid and susceptible to change throughout the admission and depending on who is leading/involved in the child's care at any one point. These changing levels of satisfaction appear to feed into and form the contexts within which the ongoing care takes place. The post-discharge satisfaction outcome may be more 'fixed', and this will influence any future acute care episodes.
Within your contexts – it isn't clear which concepts are patient, family, or provider based. As you mentioned in the text – contextual factors include individual factors (would be both the family and patient), provider factors and organizational.	The initial programme theory provides a very basic overview of how the acute care outcomes for CCCN and their families may be achieved. At present we do not know enough about the acute care for CCCN to know whether the contexts are specific to particular stakeholders or applicable to all of them. The review will help us to better

	understand these contexts and which stakeholders they are relevant to.
Are you going to be focusing on a certain type of provider or any type of intervention provided in the acute care setting by any type of provider?	This review will focus on acute care delivered by any acute care provider. This has been clarified in Table 1.
In your initial programme theory under 'what are the mechanisms?' box you have 'feeling valued' and 'feeling cared for'. Aren't those outcomes? Also are you conceptualizing partnership as a mechanism or is that an outcome? There are many mechanisms by which providers engage families in the care of the pediatric patient that are not reflected in your initial programme theory. The only things labeled as mechanisms right now are 'trust', 'respect', 'participation', 'feeling valued', and 'feeling cared for'. Maybe I am misinterpreting the model?	As this is only an initial programme theory, we do not have a list of CMOC's available but have briefly developed two below which will hopefully explain how we believe some of the concepts are interchangeable in how they function as contexts, mechanisms and outcomes, within different CMOCs.<br>As you righty identify, 'feeling cared for' could be an outcome within a CMOC. However, it could also function as a mechanism in another CMOC e.g.<br><br>"If health professionals provide adequate emotional and practical support to parents (C), then it will facilitate partnership working (O), because parents feel cared for/supported (M)."<br><br>This partnership outcome could be a process outcome which then feeds into another CMOC e.g.<br><br>"If families feel that they are working in partnership with health professionals (C), then they will have better satisfaction with care (O) because they trust (M) the team caring for their child".<br><br>It is likely that some of the concepts outlined in the IPT diagram function as context, mechanisms and outcomes in different CMOCs and in different stages/parts of the programme theory.<br><br>At this stage we have provided a very generalised overview of how acute care for CCCN may work to achieve optimal outcomes. We are at this stage uncertain of what the relevant mechanisms are and the box in the diagram provides a few suggestions of possible mechanisms. The review is now necessary to find the data to build the CMOCs.
Please be explicit about who was responsible for constructing the initial programme	Clarification added (in "Step 1: Clarifying the scope and review question") that EP

theory.	developed the programme theory and that it was refined through discussion with the other authors and PPIE advisors.
**Reviewer 3**	
The initial programme theory diagram is a little hard to read and my only suggestion is to optimise this - though on a full A4 page in print it may be less problematic.	Thank you for highlighting this. We have made minor changes to the font on the diagram which we hope makes it more readable. We expect that a larger version will be available when published which will make it easier to view.

VERSION 2 - REVIEW

Reviewer 2

Name Jerofke-Owen, Teresa

Affiliation Marquette University - College of Nursing

Date 10-Feb-2025

COI

I commend you for your attention and responses to the comments provided on the initial review. I think the protocol reads much more smoothly and I have no further comments or concerns.
